# Phytochemical Screening and Antiprotozoal Effects of the Methanolic *Berberis Vulgaris* and Acetonic *Rhus* *Coriaria* Extracts

**DOI:** 10.3390/molecules25030550

**Published:** 2020-01-27

**Authors:** Gaber El-Saber Batiha, Amany Magdy Beshbishy, Oluyomi Stephen Adeyemi, Eman Hassan Nadwa, Eman kadry Mohamed Rashwan, Luay M. Alkazmi, Amr A. Elkelish, Ikuo Igarashi

**Affiliations:** 1 National Research Center for Protozoan Diseases, Obihiro University of Agriculture and Veterinary Medicine, Nishi 2-13, Inada-cho, Obihiro 080-8555, Hokkaido, Japan; amanimagdi2008@gmail.com (A.M.B.);; 2 Department of Pharmacology and Therapeutics, Faculty of Veterinary Medicine, Damanhour University, Damanhour 22511, AlBeheira, Egypt; 3 Department of Biochemistry, Medicinal Biochemistry, Nanomedicine and Toxicology Laboratory, Landmark University, Omu-Aran 251101, Kwara State, Nigeria; oluyomiadeyemi@gmail.com; 4 Department of Pharmacology and Therapeutics, College of Medicine, Jouf University, Sakaka 72345, Saudi Arabia; 5 Department of Medical Pharmacology, Faculty of Medicine, Cairo University, Giza 12613, Egypt; 6 Department of Physiology, College of Medicine, Al-Azhar University, Assuit 71524, Egypt; dremanrashwan2020@gmail.com; 7 Department of Physiology, College of Medicine, Jouf University, Sakaka 42421, Saudi Arabia; 8 Biology Department, Faculty of Applied Sciences, Umm Al-Qura University, Makkah 21955, Saudi Arabia; lmalkazmi@uqu.edu.sa; 9 Department of Botany, Faculty of Science, Suez Canal University, Ismailia 41522, Egypt; amr.elkelish@science.suez.edu.eg

**Keywords:** *Berberis vulgaris*, *Rhus coriaria*, *Babesia*, *Theileria*, drug candidates, clinical studies, pharmacological activities

## Abstract

*Berberis vulgaris* (*B. vulgaris*) and *Rhus* *coriaria* (*R.* *coriaria)* have been documented to have various pharmacologic activities. The current study assessed the in vitro as well as in vivo inhibitory efficacy of a methanolic extract of *B. vulgaris* (MEBV) and an acetone extract of *R.* *coriaria* (AERC) on six species of piroplasm parasites. The drug-exposure viability assay was tested on three different cell lines, namely mouse embryonic fibroblast (NIH/3T3), Madin-Darby bovine kidney (MDBK) and human foreskin fibroblast (HFF) cells. Qualitative phytochemical estimation revealed that both extracts containing alkaloid, tannin, saponins and terpenoids and significant amounts of flavonoids and polyphenols. The GC-MS analysis of MEBV and AERC revealed the existence of 27 and 20 phytochemical compounds, respectively. MEBV and AERC restricted the multiplication of *Babesia* (*B.*) *bovis*, *B. bigemina*, *B. divergens, B. caballi*, and *Theileria* (*T.*) *equi* at the half-maximal inhibitory concentration (IC_50_) of 0.84 ± 0.2, 0.81 ± 0.3, 4.1 ± 0.9, 0.35 ± 0.1 and 0.68 ± 0.1 µg/mL and 85.7 ± 3.1, 60 ± 8.5, 90 ± 3.7, 85.7 ± 2.1 and 78 ± 2.1 µg/mL, respectively. In the cytotoxicity assay, MEBV and AERC inhibited MDBK, NIH/3T3 and HFF cells with half-maximal effective concentrations (EC_50_) of 695.7 ± 24.9, 931 ± 44.9, >1500 µg/mL and 737.7 ± 17.4, >1500 and >1500 µg/mL, respectively. The experiments in mice showed that MEBV and AERC prohibited *B. microti* multiplication at 150 mg/kg by 66.7% and 70%, respectively. These results indicate the prospects of these extracts as drug candidates for piroplasmosis treatment following additional studies in some clinical cases.

## 1. Introduction

Piroplasmosis is the exhausting ailment caused by hematotropic piroplasm parasites [1]. Up to date, numerous drugs have been used for the treatment of piroplasmosis infections [2,3]. For instance, clindamycin-quinine, pentamidine–cotrimoxazole and atovaquone (ATV) combination with either azithromycin or proguanil have been reported for zoonotic babesiosis treatment, yet some investigations reported *Babesia* (*B.*) *gibsoni* resistance towards ATV [4,5]. Additionally, diminazene aceturate (DMA), oxytetracycline and imidocarb dipropionate, the treatment choices for bovine and equine piroplasmosis showed limited efficacy and there have been reported of unwanted symptoms and the emergence of resistant parasites [6]. In general, advances in piroplasmosis treatment is vital for improving disease treatment and tick control [7,8]. Several antipiroplasmic molecules and drug targets were identified, presenting an alternative choice for disease control [4,5,6,9,10,11]. The extensive socio-economic and welfare effects of bovine and equine piroplasmosis on animals and human babesiosis on humans have sustained the demand for pharmaceutical advancements to develop novel drug candidates. 

Herbal remedies have been attracting attention as a prospective alternative resource of therapy for diverse diseases across many nations [12,13]. In recent decades, medicinal plants have been gaining wider acceptance due to the perception that these plants being natural products have lesser side effects and improved efficacy than their synthetic counterparts [9,12]. Many plant species have been reported to have pharmacological activities attributable to their phytoconstituents such as glycosides, saponins, flavonoids, steroids, tannins, alkaloids, terpenes, etc. [11,14].

*Berberis vulgaris* L. Sp. Pl. 1: 330 (-331) (1753) (*B. vulgaris*), in the genus *Berberis* (family Berberidaceae), is the most widely known *Berberis* that is indigenous to semi-tropical areas in Africa, Asia, Europe, North America, and South America and it is used mainly as food [15]. Pharmacologically, *B. vulgaris* have been documented to have several therapeutic properties, including anti-inflammatory, sedative, antipyretic, antiemetic, antioxidant, anti-cholinergic, anti-arrhythmic, anti-leishmaniasis, antimicrobial, and anti-malarial [16,17,18]. The phytochemical examination showed the existence of several molecules such as phenolic compounds, tannins, sterols, vitamins (vitamins A and C), protein, carotenoid, anthocyanin, dextrose, malic acid, fructose, citric acid, pectin, tartaric acid, resin, elements (calcium, iron, and potassium, zinc, copper, and manganese), alkaloids and triterpenes [18]. Berberine and berbamine are the most valuable alkaloids exhibiting a variety of activities, such as hypoglycemia, anti-inflammatory, hypotensive, antioxidant, and hypolipidemic properties [19,20]. Previous studies documented the berberine significance in the treatment of schistosomiasis disease incomparable to the anti-schistosomiasis drug; praziquantel and they reported the beneficial effects of berberine on *Schistosoma* (*S.*) *mansoni* by inducing oxidative stress which may be the main cause of its antioxidant activity [21]. Additionally, berberine has the same effect in *Plasmodium* (*P.*) *chabaudi* through its anti-inflammatory effect as it acts by reducing the inflammatory response induced by the parasite and increasing the activities of alanine and aspartate transaminases [22,23]. Interestingly, recent reports documented the potent antiparasitic effect of *B. vulgaris* as well as berberine against *Trichomonas vaginalis*, *Giardia lamblia, Entamoeba histolytica,* and some *Leishmania* (*L.*) spp. For instance, Mahmoudvand et al. [20] revealed that *B. vulgaris* methanolic extract showed potent leishmanicidal activity against *L. infantum* and *L. tropica* (IC_50_ = 4.83 μg/mL) in vitro. Additionally, Vennerstrom et al. [24] reported that berberine alone demonstrated a potential leishmanicidal effect with an IC_50_ value of 7.1 μM and ranging from 2.1 to 26.6 μg/mL against *L. donovani*, *L. tropica,* and *L. major* promastigotes, respectively. The root bark extract of *B. vulgaris* was used to prepare a lotion formula which led to good suppression effects in mice with 90% recovery from cutaneous leishmaniasis [25,26]. 

*Rhus coriaria* L., Sp. Pl. 1: 265 (1753) (*R. coriaria*), of the genus *Rhus* that is consisting of more than 250 kinds of flowering plants of the Anacardiaceae family, which is widely reported as a medicinal herb for wound healing in addition to its common use as a spice in the Mediterranean and the Middle East [27]. Interestingly, *R. coriaria* contains a set of biologically active compounds including volatile substances, flavonoids, tannins, αpinene, cembrene β-caryophyllene, oxygenated terpenes (β-caryophyllene alcohol, hexahydrofarnesylacetone, α-terpineol, carvacrol, farnesyl acetone, aliphatic aldehydes), 2,3,6-trihydroxy-7-hydroxymethylene xanthone-1-carboxylic acid, β-sitosterol-β-d-glucoside, 2-methoxy-4-hydroxy-7-methyl-3-*O*-β-d-glucopyranosyl xanthone-1,8-dicarboxylic acid, 2,3-dihydroxy-7-methyl xanthone, tannins, myricetin and organic acids (malic and citric acids) that exhibit many pharmacological activities such as anti-gastric, antidiarrheal, antispasmodic, antidysenteric, hepatoprotective, antihepatotoxic, heptatonic, astringent, protisticide, analgesic, anti-inflammatory, antioxidant, antiviral, antibacterial and antimalarial [28,29,30]. Moreover, Gathirwa et al. [31] reported the broad-spectrum antibacterial effect of *Rhus* organic solvent extracts toward acid-fast and spore-forming, Gram-positive and Gram-negative bacteria, and this activity may be attributed to the presence of tannin [30,31]. Pal Singh et al. [32] documented the antioxidant, antiobesity, antiasthmatic, hepatoprotective and anticancer activity of gallic acid, the main active principle of *R. coriaria*. 

Although these plants have been reported for various pharmacological values, there is no evidence on the antipiroplasmic activity of *B. vulgaris* and *R. coriaria* crude extracts. Thus, the current study examined the effectiveness of a methanolic extract of *B. vulgaris* (MEBV) and an acetone extract of *R.* *coriaria* (AERC) on the multiplication of *Theileria* (*T.*) *equi*, *B. bigemina, B. bovis*, *B. caballi* and *B. divergens* using the in vitro fluorescence assay and their chemotherapy prospects against *B. microti*-infected mice.

## 2. Results

### 2.1. Plant Extraction, Phytochemical and Total Phenolic and Flavonoid Contents Evaluation of MEBV and AERC Extracts

The yield percentage of the MEBV and AERC were 7.31 and 8.06% *w*/*w* dry matter and dark in color. Preliminary examination of MEBV pointed to the existence of different phytoconstituents such as tannins, saponins, alkaloids, and terpenoids, while AERC contains saponins, alkaloids, and terpenoids that may be responsible for their pharmacological activities. Moreover, considerable amounts of polyphenols and flavonoid contents were observed in MEBV and AERC. Notably, MEBV (63.2 ± 4.2 mg of GAE/g DW) showed the highest total phenolic content followed by AERC (59.9 ± 9.4 mg of GAE/g DW). Moreover, AERC (42.9 ± 5.1 mg of CAE/g DW) had the highest total flavonoid content followed by MEBV (39. 2 ± 4.7 mg of CAE/g DW). 

### 2.2. Gas Chromatography-Mass Spectrometry (GC-MS) Analysis

The GC-MS analysis of MEBV and AERC revealed the existence of 27 and 20 phytochemical compounds, respectively. The identified chemical composition of MEBV is shown in Table 1 and represented 27 compounds, while the identified chemical composition of AERC is shown in Table 2 and represented 20 compounds. The phytochemical compounds’ identification was established on the basis of the peak area, and retention time. The active principles with their retention time (RT) and percentage of peak area (%) are expressed in Figures S1 and S2.

### 2.3. Growth-Inhibition Efficacy of MEBV and AERC In Vitro 

**Figure 1:**
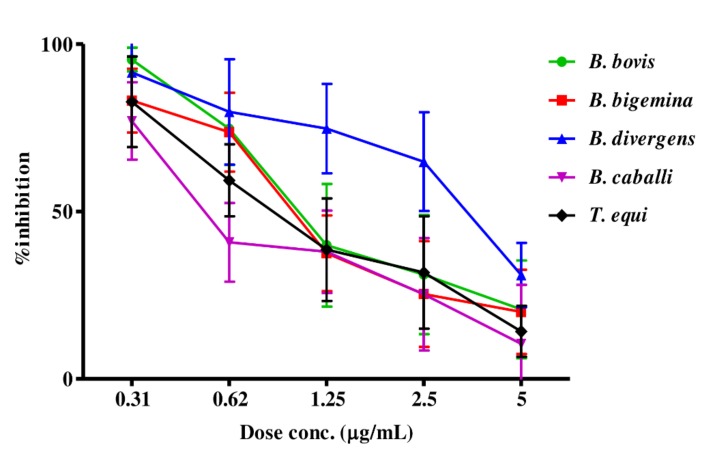
Dose-response curves of MEBV against *Babesia* and *Theileria* parasites in vitro. The curves showing the correlation between the percentage of inhibition and the concentration of MEBV (µg/mL) on *B. bovis*, *B. bigemina*, *B. divergens, B. caballi* and *T. equi*. The values obtained from three separate trials were used to determine the IC_50_’s using the non-linear regression (curve fit analysis) in GraphPad Prism software (GraphPad Software Inc., La Jolla, CA, USA).

**Figure 2:**
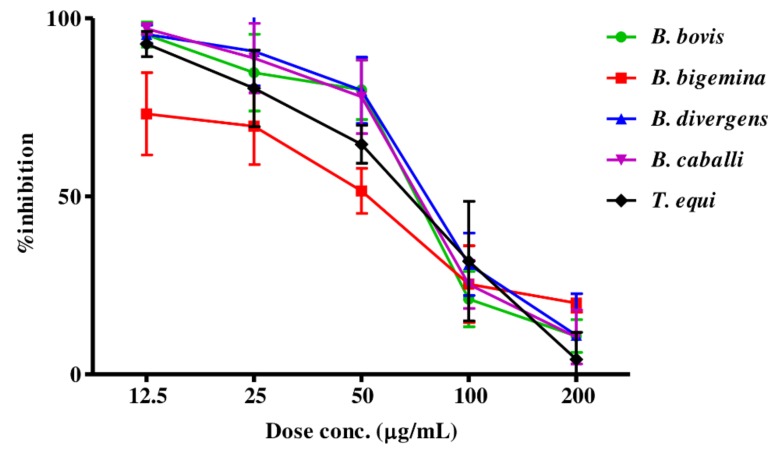
Dose-response curves of AERC against *Babesia* and *Theileria* parasites in vitro. The curves showing the correlation between the percentage of inhibition and the concentration of AERC (µg/mL) on *B. bovis*, *B. bigemina*, *B. divergens*, *B. caballi* and *T. equi*. The values obtained from three separate trials were used to determine the IC_50_’s using the non-linear regression (curve fit analysis) in GraphPad Prism software.

The preliminary evaluation of MEBV and AERC was performed to detect their efficacy on the host red blood cells (RBCs)prior to the subculture of *T. equi* and *B. bovis*. The parasite proliferation did not significantly differ between RBCs treated with either MEBV or AERC and the untreated one for both species. For the growth-inhibitory effect, MEBV (Figure 1) and AERC (Figure 2) affected the multiplication of all treated species in a dose-related manner.

MEBV and AERC suppressed *B. bovis, B. bigemina, B. divergens, B. caballi*, and *T. equi* multiplication with IC_50_s shown in Table 3. For the reference antibabesial drugs, DMA, ATV and clofazimine (CLF) restricted *B. bovis, B. bigemina, B. divergens, B. caballi*, and *T. equi* multiplication with IC_50_s shown in Table S2.

### 2.4. Parasite Viability and Morphological Changes After Treatment With MEBV and AERC

**Figure 3:**
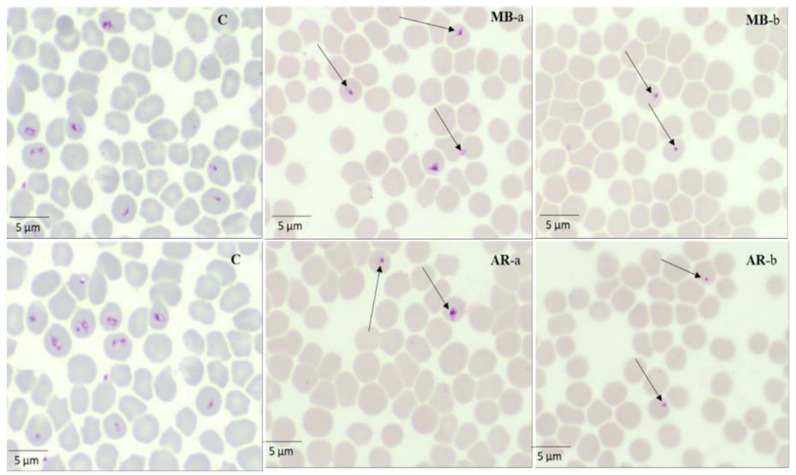
Morphological changes and light micrographs captured for MEBV- and AERC-treated *T. equi* in an in vitro culture taken after 24 (a) and 72 h (b). The arrows show the abnormal dividing parasites at 24 h. While at 72 h, drug-treated cultures showed higher numbers of degenerated parasites than did the control cultures.

In the presence of 4 × IC_50_ (16.4 and 3.24 µg/mL) MEBV, the regrowth of *B. divergens* and *B. bigemina* was completely suppressed, while *T. equi, B. caballi,* and *B. bovis* regrew even at 4 × IC_50_. AERC at 4 × IC_50_ (360, 342.8 and 312 µg/mL) completely restricted *T. equi, B. caballi* and *B. divergens* regrowth, while *B. bovis* and *B. bigemina* were able to regrow even at 4 × IC_50_. Moreover, MEBV- and AERC-treated *T. equi* micrographs are shown in Figure 3. At 24 h, all captured micrographs revealed spindle shapes dividing *B. bigemina*, *B. bovis*, and *B. caballi* parasites in contrast with the normal piriform shape of the untreated one, while dot-shaped residues of dead parasites were observed within the erythrocytes at 72 h. At 24 h, *T. equi* treated with MEBV and AERC were smaller and pyknotic when compared with the normal oval form of the untreated one. In subsequent micrographs taken at 72 h, the dying parasites appeared dot-shaped (Figure 3).

### 2.5. Toxicity of MEBV and AERC on Normal Cells 

The cytotoxicity assay of MEBV and AERC was estimated on HFF, NIH/3T3, and MDBK cell lines to detect their effect on the host cell viability. MEBV and AERC showed inhibition on MDBK and NIH/3T3 cell lines with EC_50_ shown in Table 3. Moreover, both extracts did not reduce the HFF cell viability at 1500 µg/mL. For the reference antibabesial drugs, neither DMA nor ATV (final concentration 100 µM) restricted the viability of HFF, NIH/3T3, or MDBK cell lines, while CLF in EC_50_ of 34 ± 3.4 µM reduced the MDBK cell viability (Table S2). The highest selective index values (ratio of the EC_50_ on the cell lines to the IC_50_ on the parasites) for MEBV and AERC were 1023.1 and 13.2 times toward *T. equi* and *B. bigemina*, respectively, whilst the lowest of MEBV and AERC was achieved on *B. divergens* with a selectivity index of 169.7 and on *B. divergens* with a selectivity index of 8.2, respectively (Table 3).

### 2.6. In Vitro Potential of The Combination of MEBV and AERC With Other Drugs (ATV, DMA, and CLF)

MEBV- and AERC-DMA treatment was additive toward all tested species except toward *B. caballi* it was synergetic. The combination treatments of MEBV and AERC with ATV or CLF were synergetic and additive toward all tested species (Table 4).

### 2.7. In Vivo Chemotherapeutic Efficacy of MEBV and AERC 

**Figure 4:**
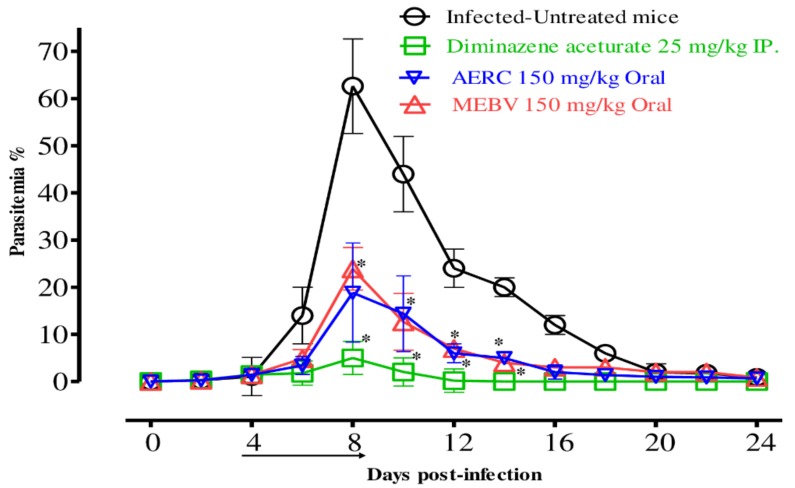
Growth inhibition effect of MEBV and AERC treatment against *B. microti* in vivo. The arrow indicates 5 consecutive days of treatment start from day 4 to 8 p.i. Asterisks indicate statistically significant (*p* < 0.05) differences of parasitemia between treated groups and the untreated group based on unpaired *t*-test analysis. Parasitemia was detected using Giemsa-stained thin blood smears by counting infected RBCs (iRBCs) among 2000 RBCs.

To examine the chemotherapeutic effects of MEBV and AERC *in vivo,* female BALB/c mice were affected by *B. microti* and the two drugs were administered for five days after the infection reach 1% parasitemia. On the eighth day post-infection (p.i.), the DDW control group showed rapid parasitemia growth reached 62.6% and the parasitemia reduced slowly on the subsequent days. The peak parasitemia level in all treated groups was 20.9%, 18.8% and 5% in MEBV (150 mg/kg BW), AERC (150 mg/kg BW) and DMA (25 mg/kg BW), respectively (Figure 4).

**Figure 5:**
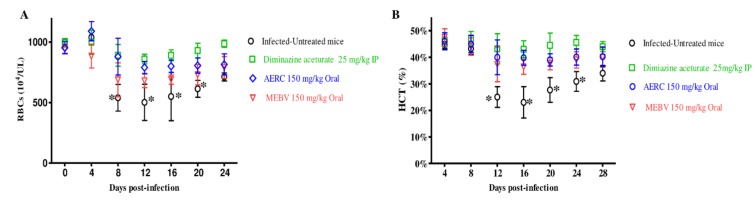

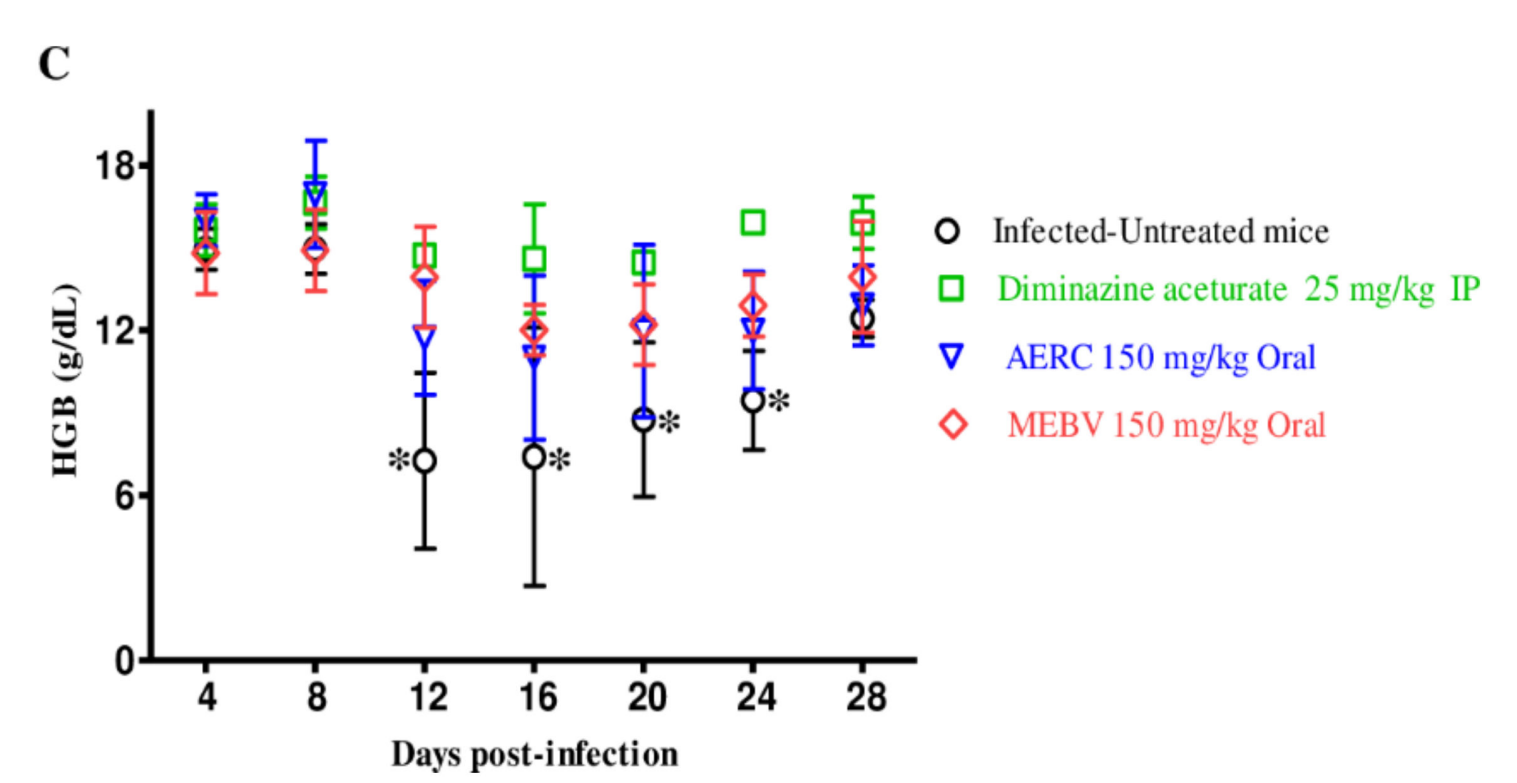
Hematology parameters changes in MEBV- and AERC-treated mice in vivo. Graphs showing the changes in RBCs count (**A**), HCT percentage (**B**), and HGB concentration (**C**) in mice treated with DMA and MEBV and AERC. Asterisks indicate statistical significance (*p* < 0.05) based on unpaired *t*-test analysis.

Moreover, the comparison of the hematology parameters during in vivo studies showed no significant difference in RBCs counts (Figure 5A), hematocrit (HCT) percentage (Figure 5B), hemoglobin (HGB) concentration (Figure 5C), or for the groups treated with MEBV as compared to the DMA-treated group on days 8 and 12. 

## 3. Discussion

In the current study, MEBV and AERC were examined for the existence of various biologically active compounds and the primary screening emphasized that both extracts containing terpenoids, alkaloids, flavonoids, and tannins. The qualitative examination revealed the presence of significant amounts of polyphenols and flavonoids. Notably, this finding conforms to the report by Gabr et al. [33] and Sequeda-Castañeda et al. [34] who revealed the existence of these active constituents in both extracts. It has been shown that all these secondary metabolites have many therapeutic properties and are known to be pharmacologically active components. Among these constituents, some flavonoids, polyphenols, and alkaloids have been reported to possess an antiparasitic effect against several parasites including, *Plasmodium*, *Leishmania*, *Trypanosoma*, *Schistosoma* and *Trichomonas Vaginalis* [35].

The phytochemical constituents of MEBV and AERC were detected using GC-MS analyses. The analysis revealed that MEBV consisted of 27 compounds, and the main chemical components identified were 3-(2-hydroxyphenyl) acrylic acid (15.55%), α-curcumene (13.27%), 3-phenyl-2-propen-1-ol, (10.16%), phenyl ethyl alcohol (9.58%), 2-methoxy-4-vinylphenol (8.41%) and 4-hydroxy-4-methyl-2-pentanone (6.43%). While AERC was found to possess 20 compounds and the main chemical components identified were 1,2,3-benzenetriol (pyrogallic acid) (21.04%), 2-butenedioic acid (21%), 1,4-*p*-menthadien-7-al (10.07%), 4-(1-methylethyl) benzaldehyde (9.33%), 4-hydroxy-4-methyl-2-pentanone (8.87%) and methyl eugenol (3.60%). Some of our GC-MS results were consistent with previous reports [18,28,29,30], however, the difference in some compounds may be attributed to the type of solvents used and the extraction method. 

For the in vitro experiments, MEBV revealed a growth-inhibiting effect against *Babesia* and *Theileria* parasites, whereas AERC exhibited high growth-inhibiting effects with high IC_50_ values against piroplasm parasites. Owing to the fact that *Babesia*, *Theileria*, and *Plasmodium* are classified into the same Apicomplexa phylum, the growth-inhibiting effect of MEBV and AERC against *Babesia* and *Theileria* parasites are consistent with those reported by Oryan. [16], and Rahimi-Madiseh et al. [18], who indicated that *B. vulgaris* extract was evaluated as a folk therapy for the treatment of fever and malaria. Additionally, Gathirwa et al. [29] showed that *R.* *coriaria* extract has antiplasmodial and antimalarial efficacy. In a separate report, the antimalarial action of *R. coriaria* was attributed to eriodictyol, 7-*O*-methylnaringenin, 7-*O*-methylluteolin, 30-*O*-dimethylquercetin, 7-*O*-methyl-apigenin, and di-*O*-methyl tetrahydroamentoflavone [30]. Further, diverse biological activities of MEBV have been attributed to the presence of berberine main active compounds [20]. Moreover, Fata et al. [25] investigated the effectiveness of alcoholic extracts of *B. vulgaris* against other parasites including cutaneous leishmaniasis. Interestingly, recent studies documented the antiprotozoal activities of various bioactive molecules identified in our GC-MS analysis. For instance, Le et al. [36] documented the antitrypanosomal activity of α-curcumene, the main chemical component in MEBV with IC_50_ value of 13.38 µg/mL against *Trypanosoma brucei brucei*, whereas Andrade et al. [37] disclosed the antileishmanial efficacy of guaiol against *Leishmania amazonensis* promastigotes forms. Moreover, Colares et al. [38] and Le et al. [39] proved antileishmanial activity of eugenol and methyleugenol (the main chemical components in AERC) against promastigotes of *L. amazonensis*. In addition to that, β-guaiene and caryophyllene show antiprotozoal activity against *P. falciparum, T. brucei, T. cruzi, L. amazonensis, and L. infantum* with IC_50_ values ranging from 2.2 to 56.6 µg/mL [40]. Sena-Lopes et al. [41] investigated the antiprotozoal effect of methyleugenol, β-caryophyllene and α-curcumene against the growth of *T. vaginalis*, with an IC_50_ value of 100 μg/mL. The GC-MS analysis of *Syzygium* (*S.*) *aromaticum* documented the presence of eugenol, β-guaiene, caryophyllene, and α–ylangene which showed potent antipiroplasmic effect in vitro and in vivo [9,42]. Therefore, we hypothesized that α-curcumene, guaiol, α-guaiene, α-ylangene, caryophyllene, and methyleugenol are the main active compounds responsible for the antipiroplasmic activity of MEBV and AERC.

It is likely that more than one bioactive ingredient restricts piroplasm parasites multiplication, thus, elucidating the inhibitory effectiveness of MEBV and AERC would require further studies. As compared to previous studies, MEBV IC_50_ values against *Babesia* parasites were less than those of S. *aromaticum* methanolic extract toward bovine *Babesia* (*B. bovis* and *B. bigemina*) [9]. While, AERC IC_50_ values toward *Babesia* parasites were less than those of *Uncaria tomentosa* acetonic extract, *Myrtus communis* acetonic extract, *Origanum vulgare* ethanolic extract, and *Cuminum cyminum* methanolic extract toward bovine *Babesia* (*B. bovis* and *B. bigemina*) [6]. A viability study revealed that MEBV and AERC have the ability to restrict piroplasm parasites regrowth. For instance, *B. caballi* and *B. divergens* treated with MEBV did not revive at 4 × IC_50_ concentration. While, AERC suppressed *B. caballi*, *T. equi* and *B. divergens* regrowth at 4 × IC_50_. However, there is a need for additional researches to isolate and identify the effective compounds from MEBV and AERC and evaluate the actual mode of action employed against the recovery of piroplasm parasites.

Additionally, MEBV and AERC treatment resulted in morphological changes in the treated parasites observed in micrographs taken at different incubation times. The captured micrographs revealed that *Babesia* and *Theileria* parasites were not able to eject and thus, died inside the iRBCs. Interestingly, recent studies revealed that the organic extract of *B. vulgaris* and *R.* *coriaria* extracts showed a significant antiparasitic effect toward *P. falciparum* and *Leishmania* with multiple morphological changes of the parasites [16,29,31]. Oryan. [16] showed that the IC_50_ of *B. vulgaris* was 45.5 µg/mL with the highest inhibitory effect on promastigotes multiplication and morphological changes, demonstrating that, the extracts prepared from *B. vulgaris* showed more promising antileishmanial activity against both stages of the parasite. Gathirwa et al. [31] showed that the high activity of *R. natalensis* and chemosuppression 83.15% for *R. coriaria* against *P. falciparum,* explained the *Rhus* plant’s potential.

The attempts to determine the cytotoxic effect of our extracts revealed that MEBV and AERC inhibited HFF, NIH/3T3, and MDBK cell viability at a slightly high selectivity index. Thus, suggesting that the tested extracts are able to act on *Babesia* and *Theileria* without affecting the host cells. These results are comparable with that of Oryan. [16], who reported the lower cytotoxicity of *B. vulgaris* against host cells (IC_50_ = 390.5 µg/mL), with an excellent selectivity index equal to 24. Gathirwa et al. [31] showed that *R. *coriaria** did not show any cytotoxic effect on mammalian cells with an EC_50_ of 211.78 µg/mL and a selective index value of 2.78. 

Nowadays, combination chemotherapies are used to treat serious ailments, including pulmonary tuberculosis, malignancy, immune deficiency syndrome, and some protozoal diseases to promote higher therapeutic efficacy [43]. The recent strategy to enhance the therapeutic effect in Chinese medicine which began a long time ago relies on the use of herb-herb and herb-compound combination [10]. MEBV and AERC combined with DMA exhibited an additive efficacy toward all species tested. The combined application of MEBV and AERC with ATV and CLF showed an additive and synergetic relationship toward *T. equi*, *B. divergens*, *B. bigemina*, *B. caballi,* and *B. bovis*. One possible explanation is that herbal extracts contain many bioactive ingredients that may interact differently resulting in synergistic action observed for combination treatment. The implication of this is that the plant extracts could be used as an adjuvant to the piroplasmosis therapy.

MEBV and AERC produced promising in vivo antibabesial activity. On the eighth-day post-infection, oral administration of 150 mg/kg of MEBV and AERC resulted in 66.7% and 70% inhibition compared to 92% inhibition showed by DMA at 25 mg/kg. MEBV exhibited chemotherapeutic efficacy higher than the 42.4% demonstrated by *Camellia sinensis* methanolic extract [9], while the inhibitory effect of AERC was higher than the 69.2% demonstrated by *S. aromaticum* methanolic extract [9]. Furthermore, neither the MEBV nor the AERC treatment showed any apparent toxic symptoms or promoted anemia in uninfected mice. The chemotherapeutical effectiveness induced by MEBV and AERC toward *B. microti* implicates that they may be alternate chemotherapy against *B. microti* infection in humans. Therefore, identifying the active compound is necessary for contriving a higher chemosuppression effect from these extracts for the future discovery of a novel potential drug against piroplasmosis. 

The limitation of this study is the use of the whole extract to confirm the antiprotozoal activity, and it is recommended to evaluate the antipiroplasmic efficacy of the GC-MS identified compounds for the future discovery of a novel potential drug against piroplasmosis. And evaluate the actual mode of action employed against the recovery of piroplasm parasites. 

## 4. Conclusions

MEBV and AERC demonstrated the in vitro and in vivo chemotherapeutic potentials against the multiplication of several piroplasm parasites both in vitro and in vivo, implying that both extracts have potential value in treating clinical diseases in animals and humans either alone or in combination with other drugs. The GC-MS analysis documented the presence of many phytochemical compounds that may be responsible for the babesicidal activities of MEBV and AERC. 

## 5. Materials and Methods

### 5.1. Ethical Statement

The in vivo studies were conducted based on the local guidelines for animal experimentation, as approved by the Obihiro University of Agriculture and Veterinary Medicine, Japan (accession numbers 28-111-2, 28-110, and 1417-2). This ethical approval was developed through the basic guidelines for the proper conduct of animal experimentation and related activities in Academic Research Institutions, Ministry of Education, Culture, Sports and Technology (MEXT), Japan.

### 5.2. Chemical Reagents

Stock solutions (100 mg (crude extract)/1 mL (DMSO) and 10 mM) in dimethyl sulfoxide (DMSO) of crude extract and DMA (Ciba-Geigy Japan limited, Tokyo, Japan), ATV and CLF (Sigma-Aldrich, Tokyo, Japan), respectively were stored at −30 °C and used for antibabesiosis evaluation. Reference drugs including DMA, CLF, and ATV were used either singly and/or in combination with the two extracts for both the in vivo and in vitro experiments. For the fluorescence assay, SYBR Green I (SGI) stain (10,000×, Lonza, Alpharetta, GA, USA) was mixed with the lysis buffer containing saponin (0.016% *w*/*v*), EDTA (10 mM), Triton X–100 (1.6% *v*/*v*), and Tris (130 mM at pH 7.5) which was filtered using a polyethersulfone (0.22 µm) and kept at 4 °C.

### 5.3. Herbal Plants

*Berberis vulgaris* and *R. coriaria* were collected from Delta, Egypt and identified by the members of the Pharmacology and Chemotherapeutics Department, Faculty of Veterinary Medicine, Damanhour University, Egypt. *B. vulgaris* and *R. coriaria* voucher specimen numbers are A0177105 (DPV) and A 0177106 (DPV), respectively. An electric dryer (Sanyo Electric Co., Ltd., Osaka, Japan) was used to dry the plants at a temperature of 30 °C, then ground using a 60–80 mm mesh to a fine powder. Subsequently, fine plant powder (100 g) was dissolved in methanol (99.8%) (Wako Pure chemical Industrial, Ltd, Osaka, Japan) or acetone (99.5%) (Nacalai Tesque, Kyoto, Japan) (50 mL) and incubated for 72 h at a temperature of 30 °C. The preparation of slurry extract was performed following the method as previously described [9,18,44] and the extracted stock (100 mg/1 mL DMSO) was stored at −30 °C and used for antibabesiosis evaluation. The obtained extracts of the MEBV and AERC were 7.31 and 8.06 g, respectively, and the yield percentage was calculated using the following Equation [45]:

$$\mathrm{Percentage}\;\mathrm{yield}\;\mathrm{of}\;\mathrm{extract}=\frac{\mathrm{Weight}\;\mathrm{of}\;\mathrm{extracted}\;\mathrm{material}}{\mathrm{Weight}\;\mathrm{of}\;\mathrm{original}\;\mathrm{plant}\;\mathrm{material}\;\mathrm{used}}\times 100$$

### 5.4. Phytochemical Examination of Plant Extracts

MEBV and AERC were examined for the existence of terpenoids, saponins, tannins, and alkaloids using several qualitative tests as previously reported elsewhere [46].

### 5.5. Determination of Total Phenolic Content

The concentration of total phenolic content (polyphenols) present in MEBV and AERC was detected using Folin-Ciocalteu (FC) reagent method as described elsewhere [47]. A volume of 0.5 mL of both extracts (1 mg/mL) was added to 1.5 mL of 10% FC reagent (diluted 1:10 with de-ionized water) and mixed for 5 min. After that, an aliquot (3 mL) of 7.5% Na_2_CO_3_ solution was added and further incubated at 30 °C for 2 h. Finally, the absorbance was calculated at 760 nm and the content of total phenolic compounds was detected from a gallic acid standard curve and expressed as mg/g gallic acid equivalent (GAE) of the dry weight of the extract (mg GAE/g DW).

### 5.6. Determination of Total Flavonoid Content

Aluminium chloride (AlCl_3_) colorimetric method was used for the examination of total flavonoid content in MEBV and AERC as previously determined [47]. Briefly, an aliquot (1 mL) of both extracts was added to 3 mL of solvent extracts, 3.8 mL of distilled water, 200 µL of 1 M potassium acetate and 200 µL of 10% AlCl_3_ and incubated for 30 min. The absorbance was measured at 420 nm and the flavonoid content was detected from a catechin standard curve and expressed as mg/g catechin equivalents of the dry weight of individual extract (mg CAE/g DW).

### 5.7. Gas Chromatography-Mass Spectrometry (GC-MS) Analysis

The chemical composition of MEBV and AERC was performed using Trace GC-ISQ mass spectrometer (Thermo Scientific, Austin, TX, USA) with a direct capillary column TG–5MS (30 m × 0.25 mm × 0.25 µm film thickness) as previously described [48]. The column oven temperature was initially held at 50 °C and then increased by 5 °C /min to 250 °C withhold 1 min then increased to 300 at rate of 30 °C /min. The injector temperatures were kept at 260 °C. Helium was used as a carrier gas at a constant flow rate of 1 mL/min. The solvent delay was 4 min and diluted samples of 1 µL were injected automatically using an AS3000 Autosampler coupled with GC in the split mode. EI mass spectra were collected at 70 eV ionization voltages over the range of *m*/*z* 50–650 in full scan mode. The ion source and transfer line were set at 250 °C and 270 °C, respectively. The components were identified by comparison of their retention times and mass spectra with those of WILEY 09 and NIST 11 mass spectral databases.

### 5.8. Parasites and Mice

For conducting in vitro experiments, *B. bigemina* Argentine strain [49], *B. divergens* Germany strain, Texas strain *B. bovis* were cultured in cattleRBCs, whereas United States Department of Agriculture (USDA) strains *B. caballi* and *T. equi* were maintained in horse RBCs; for conducting in vivo study, eight-week-old female BALB/c mice weighing 25 grams (g) were obtained from CLEA Japan and infected with rodent-borne parasitic nonzoonotic agent, *B. microti* Munich strain (AB071177) [49,50,51]. All parasites were incubated and maintained at 37 °C in a humidified chamber under 90% N_2_, 5% O_2_ and 5% CO_2_ atmosphere using a microaerophilic stationary-phase culture [5]. 

### 5.9. Culture Conditions

GIT medium replenished with 40% horse serum was used as a growth medium for *B. caballi* parasite culture, while *T. equi* was grown in medium 199 (M199) supplemented with hypoxanthine (MP Biomedicals, Santa Ana, CA, USA; final concentration 13.6 μg/mL). *Babesia divergens* was preserved in Roswell Park Memorial Institute 1640 (RPMI 1640; Sigma-Aldrich, Tokyo, Japan) medium, replenished with 40% cattle serum and culture medium M199 was used as a growth medium for *B. bovis* and *B. bigemina* replenished with 40% cattle serum. To ensure free-bacterial contamination, all medium was supplemented with amphotericin B (0.15 μg/mL) (Sigma-Aldrich, St. Louis, MO, USA), streptomycin (60 U/mL) and penicillin G (60 U/mL). 

### 5.10. The Inhibition Assay of MEBV and AERC In Vitro

The *Babesia* fluorescent assay was carried out on the in vitro culture as previously reported elsewhere [5,52,53]. RBCs’ stock supply at 1% parasitemia was prepared by diluting parasite-iRBCs with uninfected RBCs. Briefly, in three separate trials, using two-fold dilution, different concentrations of MEBV, AERC, ATV, CLF, and DMA were prepared in the culture medium and added in 96-well plates in triplicate with 1% parasitemia and 2.5% HCT for *B. bigemina* and *B. bovis* and 5% HCT for *T. equi*, *B. caballi* and *B. divergens*. The positive control had iRBCs with final concentration of 0.3% of DMSO, whereas uninfected RBCs and the culture medium acted as the negative control. Afterward, parasite cultures were cultivated for 4 consecutive days without changing medium at 37 °C humidified multi-gas incubator in 90% N_2_, 5% O_2_, and 5% CO_2_ atmosphere. On day four of culture, an aliquot of lysis buffer (100 µL) was mixed with 0.2 μL/mL SG1 and gently added per well; subsequently, it was covered with aluminum foil to prevent exposure to light. After a 6-h incubation at 37 °C, fluorescence readings were acquired on a spectrofluorimeter (Fluoroskan Ascent, Thermo Fisher Scientific, Oceanside, CA, USA) with an excitation wavelength of 485 nm and an emission wavelength of 518 nm. 

### 5.11. Parasite Viability Test In Vitro and Morphological Changes 

The viability studies were monitored via microscopy as described previously [4,9,52]. Briefly, an aliquot (20 µL) of infected RBCs (1% parasitemia) was cultivated in 200 µL of media containing various concentrations of MEBV and AERC for 4 successive days, changing media daily. The concentrations used in this experiment were 0.25×, 0.5×, 1×, 2×, and 4× the IC_50_. On the fifth day, a mixture of iRBCs (6 µL) from each well and fresh equine or bovine RBCs (14 µL) was transferred to another plate, cultured in a medium free from drug and then left for an additional six days. The total parasite clearance was recorded as negative, while the relapse of parasites was recorded as positive. 

### 5.12. Evaluation of The Impacts of MEBV and AERC on RBCs of Host

The hemolytic efficacy of MEBV and AERC on cattle and horse RBCs was evaluated in vitro as previously reported elsewhere [51]. Initially, MEBV and AERC at a concentration of 300 µg/mL were cultivated at 37 °C with purified bovine and equine RBCs for 3 h. Afterward, the pretreated-RBCs were added to *T. equi*- and *B. bovis*-iRBCs after washing thrice with phosphate-buffered saline (PBS) to achieve 1% parasitemia. Thereafter, using a 24-well plate, an aliquot of iRBCs (100 μL) was mixed with culture media (900 μL); the control RBCs were left untreated. To monitor the parasitemia and any side effects due to the pretreatment, Giemsa-stained smears were prepared every 24 h for four days.

### 5.13. In Vitro Efficacy of The Drug Combination Treatment 

The combined efficacy of MEBV and AERC with DMA or CLF or ATV was examined using the fluorescence inhibition assay as reported previously elsewhere [4,9,52,53]. Briefly, two-drug combinations of MEBV and AERC with DMA or CLF or ATV at five selected concentrations 0.25×, 0.5×, 1×, 2×, and 4× the IC_50_ (Table S1) were added in 96 well-plates in duplicate. The drug cultivation and the fluorescence values were detected after the addition of 2×SGI mixed with lysis buffer to each well of the 96-well plate as described above. CompuSyn software was used for combination index (CI) values calculation and the synergetic degree was established as the average weighted CI values by using the following formulae; ((1 × IC_50_) + (2 × IC_75_) + (3 × IC_90_) + (4 × IC_95_))/10) and the resulted values were demonstrated using the recommended CI scale; lower than 0.90 was considered synergetic, between 0.90–1.10 was considered additive, while higher than 1.10 was considered antagonistic developed previously [53,54].

### 5.14. Cultures of Normal Cell Lines

Cultures of Human foreskin fibroblast (HFF; HFF-1 ATCC® SCRC-1041™), Madin–Darby bovine kidney (MDBK; ECACC) and mouse embryonic fibroblast (NIH/3T3; ATCC® CRL-1658™) cell lines were retrieved from −80 °C stock and cultured continuously at 37 °C under atmosphere 5% CO_2_ in our laboratory. The NIH/3T3 and HFF cell lines were maintained in Dulbecco Modified Eagle’s Medium (DMEM; Gibco, Grand Island, NY, USA), while MDBK cell line grown in Minimum Essential Medium Eagle (MEM; Gibco). Each medium was treated with 1% glutamine, 10% fetal bovine serum and 0.5% penicillin/streptomycin (Gibco). Every 72 to 96 h, the medium was replaced, and once 80% confluence was reached, the cell collection was performed by sub-culture protocol. To confirm the absence of mycoplasma contamination, 4,6-diamidino-2-phenylindole dihydrochloride (Sigma-Aldrich) stain was used [9]. 

### 5.15. Cytotoxicity Assay of MEBV and AERC on Normal Cells

The cell viability test was conducted in a 96-well plate as described elsewhere [4,9,51,52]. Briefly, an aliquot of (100 µL) cells was implanted at a concentration of 5 × 10^4^ cells/mL in DMEM or MEM with fetal bovine serum and incubated overnight under atmosphere 5% CO_2_ at 37 °C for attachment. Using two-fold dilutions, aliquots (10 µL) were added in triplicate to each well to attain final concentrations of 15.8 to 1500 µg/mL and incubated for an additional 24 h. The positive control wells containing cells mixed with the medium in 0.4% DMSO, whereas the negative control wells containing culture medium only. After a 24-h incubation, Cell Counting Kits-8 (CCK-8) (10 µL) was added per well followed by the incubation of the plate for another 3 hours and a microplate reader was used to measure the absorbance at 450 nm.

### 5.16. In Vivo Chemotherapeutic Effects of MEBV and AERC 

MEBV and AERC were examined for their in vivo chemotherapeutic efficacy using *B. microti*–infected BALB/c mice according to a procedure described elsewhere [49,51]. Briefly, twenty-five mice were placed in an environment free from pathogens with 22 °C temperature and adjusted humidity and under 12 h light and 12 h darkness and randomly distributed into five groups. All mice in groups 2 through 5 obtained 500 µL of 1 × 10^7^ *B. microti*-iRBC by intraperitoneal (i.p.) injection. Group 1 served as a negative control and was neither infected nor treated. At 1% parasitemia, drug treatment of the mice by i.p. continuing for five days. Group 2 act as a positive control group and received 95% double-distilled water (DDW) and 5% DMSO. Groups 3 and 4 administered 150 mg/kg body weight (BW) of MEBV and AERC by the oral route, respectively. While group 5 received 25 mg/kg BW of DMA to act as a reference to drug control. The drug administration lasted for five days starting from the fourth day to the eighth day post-infection (p.i.), and parasitemia was monitored by preparing Giemsa-stained blood smears every two days in about 5000 RBCs by microscopy until day 32 p.i. Furthermore, the hematological parameters, including HGB, RBCs, and HCT percentage were examined every four days using an automatic hematology analyzer (Celltac α MEK-6450, Nihon Kohden, Tokyo, Japan) to detect the effect of MEBV and AERC on the retrogression of anemia related to *Babesia* infection. At the end of the in vivo experiment, an anesthetic system using an inhaler containing isoflurane was used to euthanize all mice by placing them in the induction chamber, adjusting the oxygen flowmeter to 0.8 to 1.5 L/min and vaporizer to 3% to 5%. When mice were completely anesthetized, all of them were killed by cervical dislocation according to the ethical approval confirmed by the Basic Guidelines for Proper Conduct of Animal Experiment and Related Activities in Academic Research Institutions, the Ministry of Education, Culture, Sports and Technology (MEXT), Japan.

### 5.17. Statistical Analysis 

The nonlinear regression curve fit on a GraphPad Prism (GraphPad Software Inc., USA) was used to determine the IC_50_ values of MEBV, AERC, DMA, CLF, and ATV from in vitro growth of the parasites. The significant variations (*p* < 0.05) among group mean values on parasitemia and Student’s *t*-test, available in the GraphPad Prism software was used to analyze hematology profiles in mice infected with *B. microti*. 

## Figures and Tables

**Table 1:** The chemical composition of MEBV by GC-MS.

Peak	R.t	Name	Area (%)	Molecular Weight	Molecular Formula
1	8.71	2-Pentanone, 4-Hydroxy-4-Methyl-	6.43	116	C_6_H_12_O_2_
2	10.30	Propanal, 3-Ethoxy-	0.50	102	C_5_H_10_O_2_
3	13.83	*trans*-2-Pentenoic acid	0.52	100	C_5_H_8_O_2_
4	14.99	Acetic acid, Phenyl ester	0.22	136	C_8_H_8_O_2_
5	15.07	6-Nonynoic acid	0.37	154	C_9_H_14_O_2_
6	16.99	Benzyl alcohol	1.22	108	C_7_H_8_O
7	18.11	Benzene, 1,3,5-Trimethyl-	0.37	120	C9H12
8	18.57	Phenol, 2-Methoxy	1.24	124	C_7_H_8_O_2_
9	19.94	Phenylethyl Alcohol	9.58	122	C_8_H_10_O
10	20.68	Ribitol	0.90	152	C_5_H_12_O_5_
11	24.48	3-(2-Hydroxyphenyl) acrylic acid	15.55	164	C_9_H_8_O_3_
12	25.24	Octadecanoic acid, 3-hydroxy-, methyl ester	2.66	314	C_19_H_38_O_3_
13	25.74	α-Ylangene	0.39	204	C_15_H_24_
14	26.70	7,10-Pentadecadiynoic acid	0.65	234	C_15_H_22_O_2_
15	27.01	2-Methoxy-4-vinylphenol	8.41	150	C_9_H_10_O_2_
16	27.49	α -Longipinene	1.27	204	C_15_H_24_
17	27.74	2-Propen-1-Ol, 3-Phenyl	10.16	134	C_9_H_10_O
18	29.84	α-Curcumene	13.27	204	C_15_H_24_
19	30.08	*trans*-Sesquisabinene hydrate	2.50	222	C_15_H_26_O
20	30.21	α-Bulnesene	2.41	204	C_15_H_24_
21	30.67	Sesquicineole	1.07	222	C_15_H_26_O
22	31.41	2-Propen-1-Ol, 3-Phenyl-, Acetate	3.94	176	C_11_H_12_O_2_
23	31.69	Aromadendrene oxide	2.27	220	C_15_H_24_O
24	33.17	Methyl 5,7-hexadecadiynoate	3.22	262	C_17_H_26_O_2_
25	34.21	Guaiol	5.93	222	C_15_H_26_O
26	35.56	α-Guaiene	2.54	204	C_15_H_24_
27	35.76	*cis*-5,8,11,14,17-Eicosapentaenoic acid	1.30	302	C_20_H_30_O_2_

R.t, retention time (min).

**Table 2:** The chemical composition of AERC by GC-MS.

Peak	R.t	Name	Area (%)	Molecular Weight	Molecular Formula
1	8.66	2-Pentanone, 4-Hydroxy-4-Methyl-	8.87	116	C_6_H_12_O_2_
2	12.15	2-Butenedioic acid	21	116	C_4_H_4_O_4_
3	14.34	3-Thujanol	1.14	154	C_10_H_18_O
4	14.93	4-Heptenal	1.20	112	C_7_H_12_O
5	15.11	2,4-Heptadienal, (*E*,*E*)	2.12	110	C_7_H_10_O
6	17.00	2-Nonen-1-ol, (*E*)	1.00	142	C_9_H_18_O
7	19.96	4-Penten-2-Ol, 3-Methyl-	0.90	100	C_6_H_12_O
8	23.28	2-Decenal, (*E*)-	1.69	154	C_10_H_18_O
9	24.03	Benzaldehyde, 4-(1-methylethyl)-	9.33	148	C_10_H_12_O
10	25.31	α-Ylangene	0.95	204	C_15_H_24_
11	25.58	1,4-p-Menthadien-7-al	10.07	150	C_10_H_14_O
12	25.90	2,4-Decadienal, (*E*,*E*)-	1.42	152	C_10_H_16_O
13	26.68	2-Undecenal, *E*-	2.34	168	C_11_H_20_O
14	27.34	Caryophyllene	2.05	204	C_15_H_24_
15	29.50	Methyleugenol	3.60	178	C_11_H_14_O_2_
16	29.78	Benzene,1-(1,5-Dimethyl-4-Hexenyl)-4-Methyl-	1.55	202	C_15_H_22_
17	30.06	2,6,10-Dodecatrien-1-ol,3,7,11-trimethyl-	0.70	222	C_15_H_26_O
18	30.82	7-epi-*cis*-Sesquisabinene hydrate	3.47	222	C_15_H_26_O
19	31.84	1,2,3-Benzenetriol (Pyrogallic acid)	21.04	126	C_6_H_6_O_3_
20	34.70	*cis*-Z-**α**-Bisabolene epoxide	2.88	220	C_15_H_24_O

R.t, retention time (min).

**Table 3:** IC_50_ and selective index values of MEBV and AERC.

Crude Extracts	Parasites	IC_50_ (µg/mL) ^a^	EC_50_ (µg/mL) ^b^			Selective Indices ^c^		
			MDBK	NIH/3T3	HFF	MDBK	NIH/3T3	HFF
**MEBV**	***B. bovis***	0.84 ± 0.2	695.7 ± 24.9	931 ± 44.9	>1500	828.2	1108.3	>1785.7
	***B. bigemina***	0.81 ± 0.3				858.9	1149.3	>1851.9
	***B. divergens***	4.1 ± 0.9				169.7	227.1	>365.9
	***B. caballi***	0.35 ± 0.1				1987.7	2660	>4285.7
	***T. equi***	0.68 ± 0.1				1023.1	1369.1	>2205.9
**AERC**	***B. bovis***	85.7 ± 3.1	737.7 ± 17.4	>1500	>1500	8.6	>17.5	>17.5
	***B. bigemina***	55.7 ± 2.7				13.2	>26.9	>26.9
	***B. divergens***	90 ± 3.7				8.2	>16.7	>16.7
	***B. caballi***	85.7 ± 2.1				8.6	>17.5	>17.5
	***T. equi***	78 ± 2.1				9.5	>19.2	>19.2

^a^ Half-maximal inhibitory concentration of extracts on piroplasm parasites in vitro. ^b^ Half-maximal effective concentration of extracts on the tested cell lines. The dose-response curve using nonlinear regression (curve fit analysis) was used to detect all of these values. The values obtained from the means of triplicate experiments. ^c^ Selective index calculated as the ratio of the EC_50_ of cell lines to the IC_50_ of each parasite.

**Table 4:** Combination effect of MEBV and AERC with DMA, ATV, and CLF in vitro.

Parasites	Drug Combinations ^a^	CI Values (µg/mL)				Weighted Average CI Values ^b^	Degree of Association ^c^
		IC_95_	IC_90_	IC_75_	IC_50_		
***B. bovis***	MEBV + DMA	1.972	1.003	1.000	0.978	1.08900	**Additive**
	AERC + DMA	0.502	1.099	1.282	0.996	1.05300	**Additive**
	MEBV + ATV	0.722	1.691	0.968	0.908	1.06402	**Additive**
	AERC + ATV	0.865	1.297	0.913	0.973	1.00901	**Additive**
	MEBV + CLF	0.634	1.124	1.077	0.994	1.08900	**Additive**
	AERC + CLF	0.282	1.099	1.282	0.896	0.99099	**Additive**
***B. bigemina***	MEBV + DMA	2.460	1.004	1.004	0.768	1.05520	**Additive**
	AERC + DMA	1.7133	0.907	0.823	1.001	1.00003	**Additive**
	MEBV + ATV	0.9162	1.072	1.026	0.925	1.05402	**Additive**
	AERC + ATV	1.2597	0.827	0.889	0.85	0.89807	**Synergism**
	MEBV + CLF	0.578	0.872	0.926	0.725	0.80000	**Synergism**
	AERC + CLF	1.029	0.777	0.887	0.884	0.87801	**Synergism**
***B. divergens***	MEBV + DMA	1.882	0.977	0.979	1.008	1.08050	**Additive**
	AERC + DMA	1.0326	0.878	1.038	1.132	1.04306	**Additive**
	MEBV + ATV	1.946	0.897	0.987	1.002	1.07090	**Additive**
	AERC + ATV	0.8193	0.981	1.009	1.073	1.01003	**Additive**
	MEBV + CLF	1.3592	0.908	0.989	0.892	0.97102	**Additive**
	AERC + CLF	1.5631	0.991	1.091	1.093	1.11901	**Additive**
***B. caballi***	MEBV + DMA	1.443	1.095	1.235	0.908	1.09701	**Additive**
	AERC + DMA	0.573	0.092	0.072	0.002	0.09809	**Synergism**
	MEBV + ATV	1.302	1.082	1.102	1.009	1.08083	**Additive**
	AERC + ATV	0.589	0.557	0.891	0.656	0.70003	**Synergism**
	MEBV + CLF	0.557	0.787	0.787	0.577	0.68001	**Synergism**
	AERC + CLF	0.230	0.281	0.201	0.153	0.20073	**Synergism**
***T. equi***	MEBV + DMA	1.337	0.992	0.897	1.122	1.05003	**Additive**
	AERC + DMA	1.020	1.003	0.972	1.017	1.00104	**Additive**
	MEBV + ATV	1.330	0.839	0.998	1.142	1.05703	**Additive**
	AERC + ATV	0.086	0.191	0.098	0.059	0.09975	**Synergism**
	MEBV + CLF	1.044	0.309	0.387	0.474	0.47190	**Synergism**
	AERC + CLF	0.4235	0.780	0.891	0.859	0.80925	**Synergism**

^a^ Two-drug combination between MEBV and AERC with DMA, CLF, and ATV at a concentration of approximately 0.25×, 0.5×, IC_50_, 2×, and 4× the IC_50_ (constant ratio). ^b^ The weighted average CI value was calculated with the formula [(1 × IC_50_) + (2 × IC_75_) + (3 × IC_90_) + (4 × IC_95_)]/10. ^c^ The degree of synergism was determined based on the following CI value: < 0.90 (synergism), 0.90–1.10 (additive), and > 1.10 (antagonism). CI value, combination index value; IC_50_, 50% inhibition concentration; DMA, diminazene aceturate; ATV, atovaquone; CLF, clofazimine.

## Supplementary Materials

The following are available online. **Table S1.** The concentrations of the in vitro combination treatment of methanolic *B. vulgaris* and acetonic *R. coriaria* extracts with ATV, DMA, and CLF toward piroplasm parasites. **Table S2.** The IC_50_ and selective indexes value of DMA, CLF, and ATV. **Figure S1.** Gas chromatography-mass spectrometry analysis in the methanolic extract of *Berberis vulgaris.* **Figure S2.** Gas chromatography-mass spectrometry analysis in the acetonic extract of *Rhus coriaria.*
