# Supplementary figures and images for: Phytochemical Screening and Antiprotozoal Effects of the Methanolic Berberis Vulgaris and Acetonic Rhus Coriaria Extracts
Source: Molecules. 2020 Jan 27;25(3):550. doi: 10.3390/molecules25030550 (PMC7037273; doi:10.3390/molecules25030550)

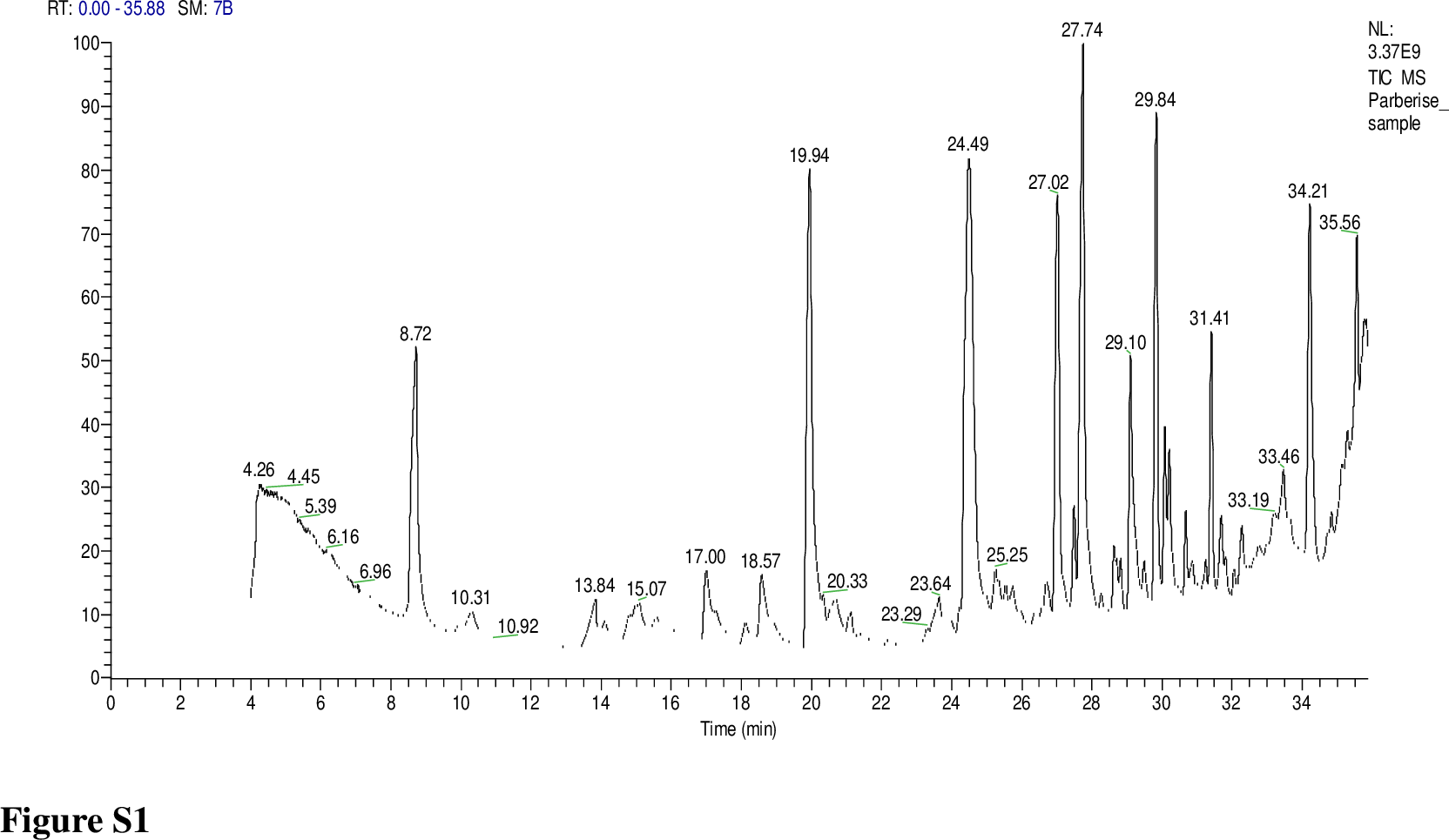

Supplement: Supplementary file 1 [file molecules-25-00550-s001.zip › Supplementary figure- S1.tif]

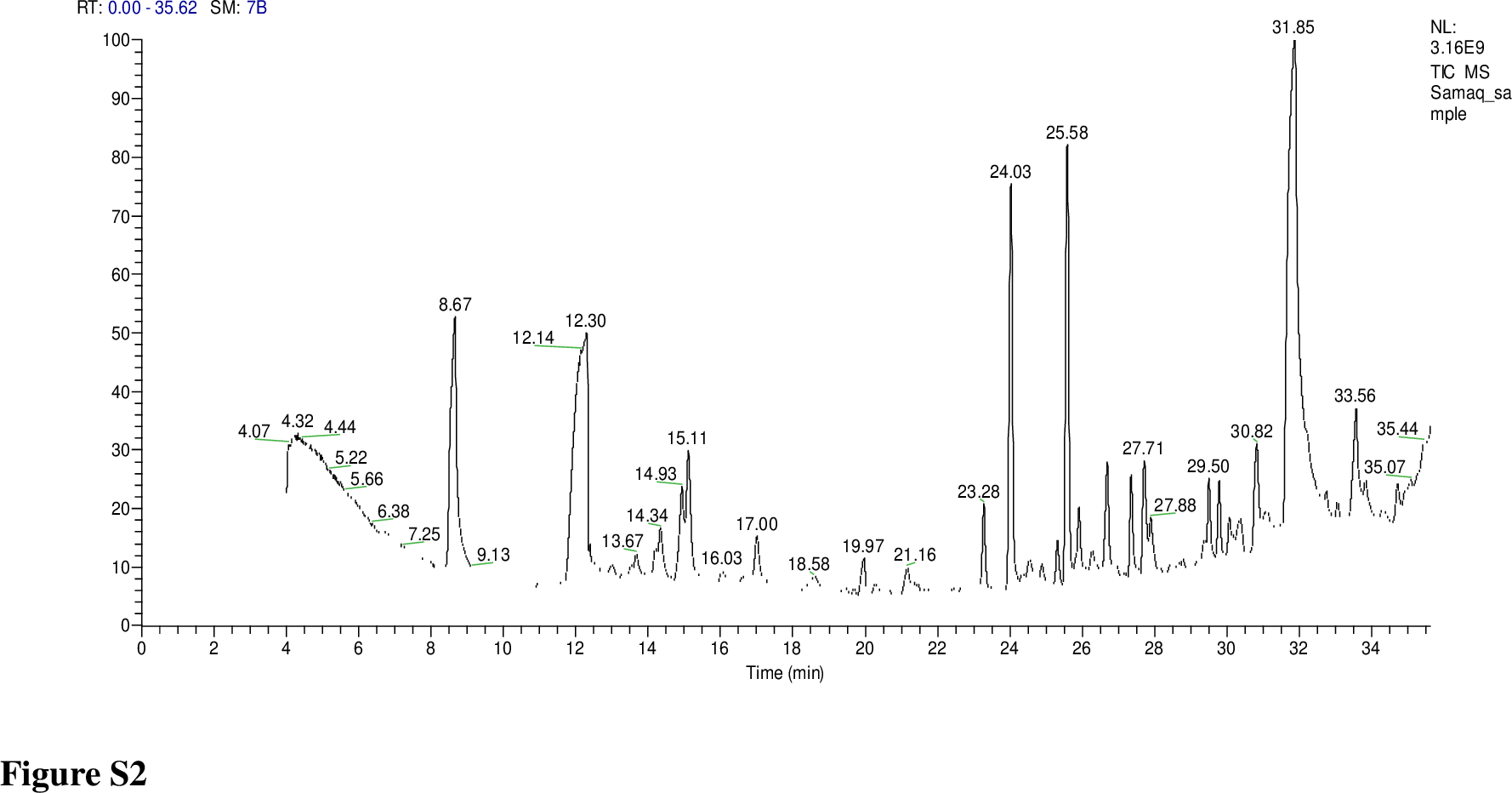

Supplement: Supplementary file 1 [file molecules-25-00550-s001.zip › Supplementary figures -S2.tif]

## Slide 1
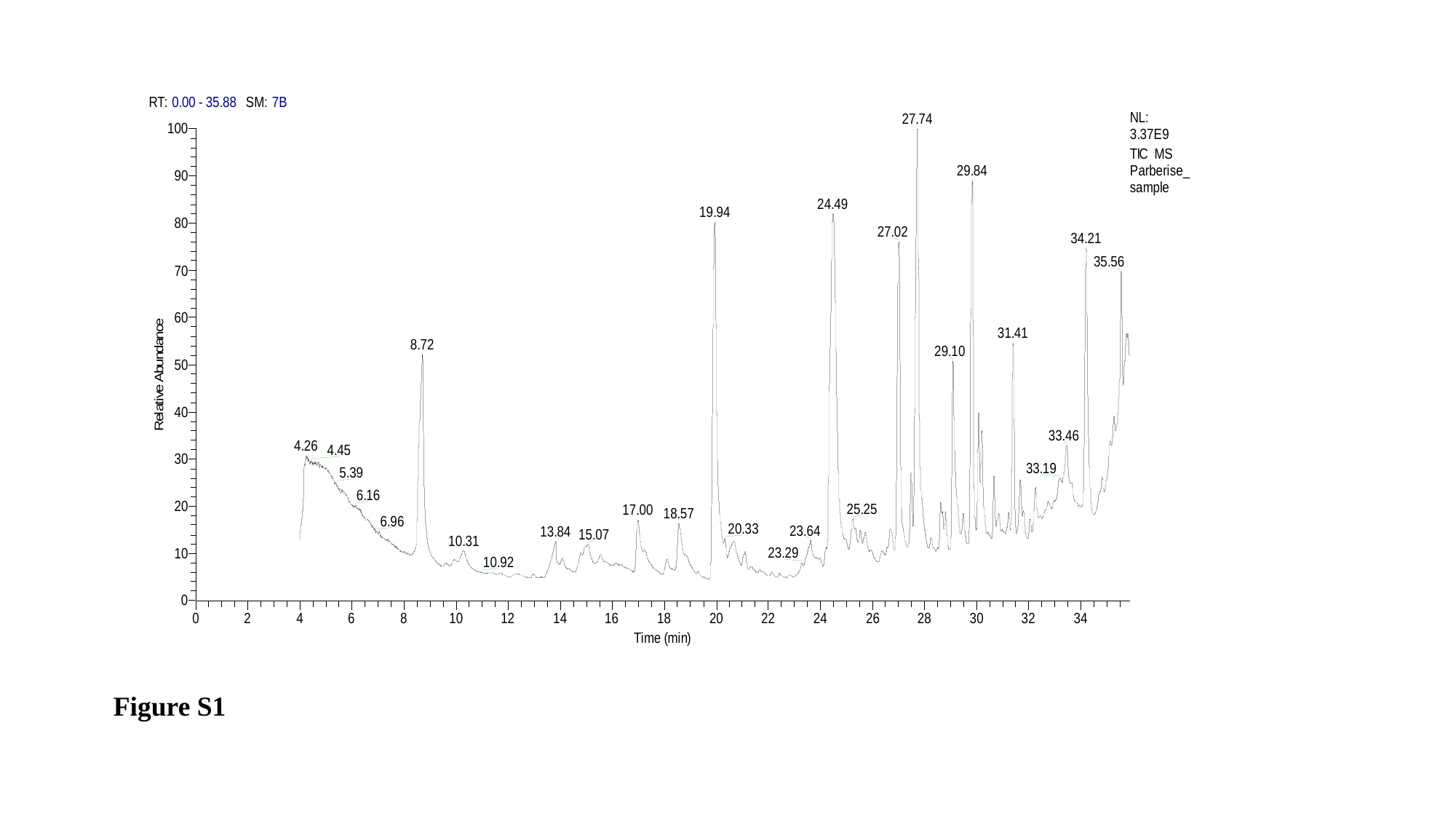

Figure S1

## Slide 2
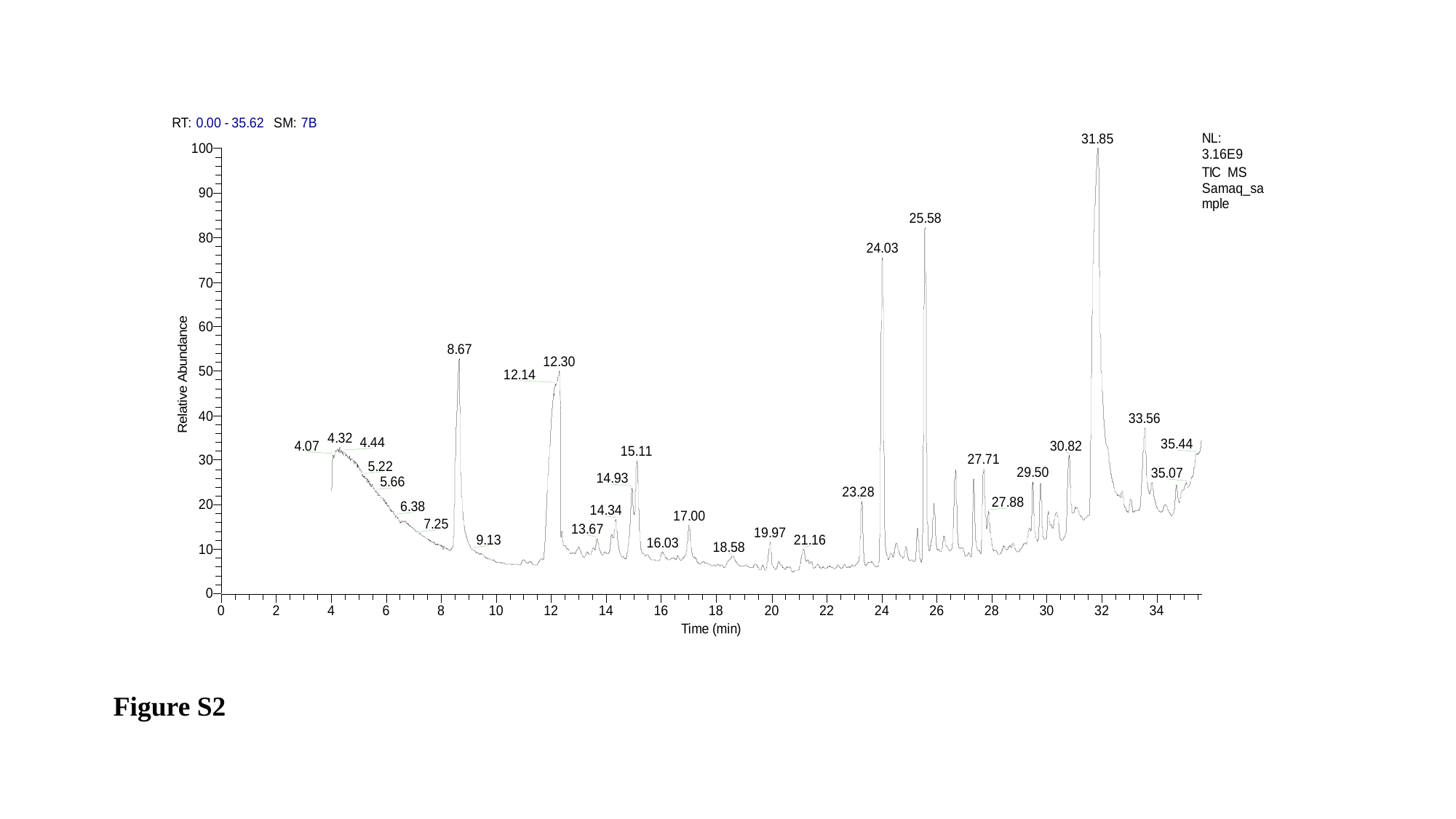

Figure S2

Supplement: Supplementary file 1 [file molecules-25-00550-s001.zip › Supplementary figures.pptx]
